# Analysis of Left Ventricular Indexes and Mortality Among Asian Adults With Hemodynamically Significant Chronic Aortic Regurgitation

**DOI:** 10.1001/jamanetworkopen.2023.4632

**Published:** 2023-03-24

**Authors:** Li-Tan Yang, Chien-Chang Lee, Chin-Hua Su, Masashi Amano, Yosuke Nabeshima, Tetsuji Kitano, Chieh-Mei Tsai, Chung-Lieh Hung, Yuriko Nakaoku, Kunihiro Nishimura, Soshiro Ogata, Hao-Yun Lo, Ron-Bin Hsu, Yih-Sharng Chen, Wen-Jone Chen, Rekha Mankad, Patricia A. Pellikka, Yi-Lwun Ho, Masaaki Takeuchi, Chisato Izumi

**Affiliations:** 1 Division of Cardiology, Department of Internal Medicine, National Taiwan University Hospital, Taipei, Taiwan; 2 Department of Internal Medicine, College of Medicine, National Taiwan University, Taipei, Taiwan; 3 Telehealth Center, National Taiwan University Hospital, Taipei, Taiwan; 4 Department of Emergency Medicine, National Taiwan University Hospital, Taipei, Taiwan; 5 Center of Intelligent Healthcare, National Taiwan University Hospital, Taipei, Taiwan; 6 Department of Heart Failure and Transplantation, National Cerebral and Cardiovascular Center, Osaka, Japan; 7 Second Department of Internal Medicine, University of Occupational and Environmental Health, Kitakyushu, Japan; 8 Cardiovascular Division, Department of Internal Medicine, MacKay Memorial Hospital, Taipei, Taiwan; 9 Institute of Biomedical Sciences, MacKay Medical College, New Taipei City, Taiwan; 10 Department of Preventive Medicine and Epidemiology, National Cerebral and Cardiovascular Center, Osaka, Japan; 11 Cardiovascular Center and Division of Cardiovascular Surgery, Department of Surgery, National Taiwan University Hospital, Taipei, Taiwan; 12 Department of Cardiovascular Medicine, Mayo Clinic, Rochester, Minnesota; 13 Department of Laboratory and Transfusion Medicine, Hospital of University of Occupational and Environmental Health, School of Medicine, Kitakyushu, Japan

## Abstract

**Importance:**

Chronic hemodynamically significant aortic regurgitation (AR) is associated with excess risk of death, yet data for Asian patients are lacking, and whether Asian patients can abide by Western guidelines as to when aortic valve surgery should be performed is unknown.

**Objective:**

To assess AR presentation and cutoffs of left ventricular ejection fraction (LVEF), LV end-systolic dimension index (LVESDi), and LV end-systolic volume index (LVESVi) that are associated with risk of death in Asian patients with AR.

**Design, Setting, and Participants:**

This retrospective cohort study included consecutive patients with chronic, moderately severe to severe AR from 3 tertiary referral centers (2 in Japan and 1 in Taiwan) from June 11, 2008, through November 19, 2020, with follow-up through November 11, 2021.

**Exposures:**

Aortic regurgitation severity, graded by a comprehensive integrated approach.

**Main Outcomes and Measures:**

The primary outcome was the association between volume-derived LVEF, LVESDi, and LVESVi and all-cause death (ACD). The secondary outcome was the association of these LV indexes with cardiovascular death (CVD). Clinical and echocardiographic data were analyzed retrospectively. A de novo disk-summation method was used to derive LV volumes and volume-derived LVEF.

**Results:**

Of 1259 patients (mean [SD] age, 64 [17] years; 934 [74%] male), 515 (41%) were Japanese and 744 (59%) were Taiwanese. The median follow-up was 4.1 years (IQR, 1.56-7.24 years). The mean (SD) body surface area was 1.67 (0.21) m^2^; LVEF, 55% (11%); LVESDi, 24.7 (5.7) mm/m^2^; LVESVi, 50.1 (28.0) mL/m^2^; and indexed mid–ascending aorta size, 24.7 (5.5) mm/m^2^. Aortic valve surgery occurred in 483 patients (38%); 240 patients (19%) died during follow-up. Overall mean (SD) 8-year survival was 74% (2%). Separate multivariate models adjusted for covariates demonstrated independent associations of LVEF, LVESDi, and LVESVi with ACD (LVEF: hazard ratio [HR] per 10%, 0.80; 95% CI, 0.70-0.92; *P* = .002; LVESDi: HR, 1.04; 95% CI, 1.01-1.06; *P* = .002; LVESVi: HR per 10 mL/m^2^, 1.11; 95% CI, 1.05-1.17; *P* < .001) and CVD (LVEF: HR per 10%, 0.69; 95% CI, 0.56-0.85; *P* < .001; LVESDi: HR, 1.05; 95% CI, 1.01-1.09; *P* = .01; LVESVi per 10 mL/m^2^: HR, 1.15; 95% CI, 1.06-1.24; *P* < .001). In the total cohort, spline curves showed that mortality started to increase for an LVEF of 53% or less, LVESDi of 22 mm/m^2^ or greater, and LVESVi of 46 mL/m^2^ or greater for both ACD and CVD. Early surgery was beneficial in 3 strata of LVESDi (<20, 20 to <25, and ≥25 mm/m^2^) and 2 strata of LVESVi (<46 and ≥46 mL/m^2^).

**Conclusions and Relevance:**

This multicenter cohort study of Asian patients with hemodynamically significant AR found cutoff values of LVEF, LVESDi, and LVESVi that were associated with increased risk of death. These findings suggest that Western guidelines seem applicable in Asian patients and, most importantly, that indexed LV parameters with a lower cutoff could be used in discriminating patients with excess mortality risk.

## Key Points

**Question:**

Are ultrasonography indexes of left ventricular ejection fraction (LVEF), LV end-systolic dimension index (LVESDi), and LV end-systolic volume index (LVESVi) associated with excess risk of death among Asian adults with hemodynamically significant chronic aortic regurgitation?

**Findings:**

In this multicenter cohort study of 1259 Japanese and Taiwanese adults, LVEF, LVESDi, and LVESVi were associated with all-cause and cardiovascular death. The cutoffs of LVEF, LVESDi, and LVESVi were 53% or less, 22 mm/m^2^ or greater, and 46 mL/m^2^ or greater, respectively, for both all-cause and cardiovascular death.

**Meaning:**

The findings suggest that use of the aforementioned LV cutoffs to guide early surgical timing may be associated with improved overall outcomes in Asian patients with aortic regurgitation.

## Teaser

This cohort study investigates associations between left ventricular (LV) ejection fraction, LV end-systolic dimension index, and LV end-systolic volume index and risk of death in Japanese and Taiwanese individuals with chronic aortic regurgitation.

## Introduction

Chronic aortic regurgitation (AR) is a common valvular heart disease (VHD), and its prevalence is projected to double before 2050 in an aging society.^1,2^ Regarding treatment of Asian patients with hemodynamically significant AR, most physicians abide by European or US VHD guidelines because most clinical evidence has been from studies conducted in Western countries.^3,4^ Although the Japanese Circulation Society developed its own VHD guidelines regarding treatment of AR,^5^ the algorithm was similar to US guidelines, with little modification based on scarce evidence originating from Japan or Asia.^6,7^

Whether current AR treatment guidelines, which largely focus on left ventricular (LV) chamber size,^3,4^ can be generalized in Asian individuals remains unknown. First, patients with Asian ancestry were underrepresented in most AR studies.^8,9,10^ Second, the cutoffs of absolute LV size—an LV end-systolic dimension (LVESD) of 50 mm and an LV end-diastolic dimension (LVEDD) of 65 mm—may fall short since Asian individuals tend to have a smaller body surface area than White individuals. Also, sociocultural factors may be associated with different outcomes in patients with AR.^11^ Some of us recently found that interethnic (eg, Western vs Asian) differences exist in patients with AR.^12^ Therefore, there is a pressing need to explore the contemporary profile of Asian patients with AR and compare it with results found among Western patients.

Japan and Taiwan are among the countries that have above-average life expectancy^13,14^ and have a compulsory health insurance system,^15^ wherein most medical expenses are covered by the government and there is ready access to medical care. In this Asian cohort with hemodynamically significant AR, we sought to (1) identify LV parameters and the cutoffs associated with risk of death; (2) explore interethnic differences, including between Asian and Western individuals and Japanese and Taiwanese individuals; and (3) explore the outcomes of aortic valve surgery.

## Methods

### Study Population and Clinical Data

In this cohort study, between June 11, 2008, and November 19, 2020, all consecutive patients aged 18 years or older with chronic moderately severe to severe AR assessed by transthoracic echocardiography (TTE) were retrospectively identified from the electronic echocardiographic database of 3 university-affiliated hospitals (National Taiwan University Hospital, Taipei, Taiwan; National Cerebral and Cardiovascular Center, Osaka, Japan; and University of Occupational and Environmental Health, Kitakyushu, Japan) (eFigure 1 in Supplement 1). Follow-up was complete by November 11, 2021. The institutional review boards at all 3 institutions approved this study; informed consent was waived because the study was retrospective. We followed the Strengthening the Reporting of Observational Studies in Epidemiology (STROBE) reporting guideline. All cases were reviewed de novo to determine eligibility. Exclusion criteria included no research authorization, more than mild mitral stenosis or regurgitation or aortic stenosis, prior mitral or aortic valve surgery, complex cyanotic congenital heart disease, carcinoid heart disease, and acute AR (dissection, trauma, or active endocarditis).

All patients had comprehensive cardiology and/or cardiovascular surgery evaluations within 30 days of TTE. Baseline symptoms, New York Heart Association (NYHA) functional class, and comorbid conditions, independently and prospectively recorded by treating physicians, were abstracted from electronic or paper medical records. Asymptomatic patients were those without typical symptoms (heart failure symptoms, exertional chest pain, dyspnea, or exercise intolerance). The Charlson Comorbidity Index (CCI) was computed. Ischemic heart disease was defined as prior myocardial infarction, coronary artery bypass graft, coronary artery disease, or history of ischemic cardiomyopathy.

### Echocardiography

In patients with multiple TTEs, the first eligible result was used as the baseline for analysis. Transthoracic echocardiography was performed by trained sonographers using commercially available echocardiography systems and reviewed de novo by cardiologists with level-3 echocardiography training. Chamber quantification was performed based on guideline suggestions.^16^ Left ventricular volumes were derived from the biplane disk-summation method or single plane if biplane was not feasible. Left ventricular filling pressure was represented by the ratio of peak mitral inflow velocity to early diastolic mitral annular velocity. For diagnosis of AR severity, an integrated, comprehensive approach was used, including quantitative (proximal isovelocity surface area [PISA] quantification) and semiquantitative measurements (vena contracta width, pressure half-time) according to guidelines.^17^ Images stored for PISA quantification were not routinely obtained in Taiwan before 2020. To ensure consistency of AR severity diagnosis, 20 randomly selected cases from the Japanese cohort were reviewed by L.T.Y. to calculate AR vena contracta; the intraclass correlation coefficient was 0.945.

### Aortic Valve Surgery and Surgical Indications

Surgical indications were stratified based on both 2017 European Society of Cardiology and 2014 American College of Cardiology guidelines^3,18^ in the following order: (1) symptoms (typical heart failure symptoms, exertional chest pain, or dyspnea), (2) LV ejection fraction (LVEF) of 50% or less, (3) LVESD greater than 50 mm or indexed LVESD (LVESDi) greater than 25 mm/m^2^, (4) surgery for aortic dilatation or aneurysms (no patients had AR of moderate or lower severity), and (5) LVEDD greater than 65 mm. Surgery status was ascertained from medical records and telephone interview, if necessary.

### Outcomes

Our primary end point was all-cause death (ACD). The secondary end point was cardiovascular death (CVD). The association between aortic valve surgery and ACD and CVD was also analyzed. Observation time was between either (1) baseline TTE and death or last follow-up (total follow-up) or (2) baseline TTE and death, time of aortic valve surgery, or last follow-up (death during medical surveillance). Mortality status, date of death, and cause of death were retrieved from medical records, mailed questionnaires, and for Taiwanese patients, the National Health Insurance Research Database. In Taiwan, the National Health Insurance program is a government-run, single-payer insurance plan that delivers universal coverage (99.5% of whole population) for all citizens and was linkable to national databases such as registries of deaths.

### Statistical Analysis

Continuous variables, expressed as mean (SD) or median (IQR) according to data distribution, were compared using the Student *t* test or Wilcoxon rank sum test as appropriate based on normality of data distribution. Categorical data, presented as counts and percentages, were compared using the χ^2^ test. Survival was estimated using the Kaplan-Meier method and compared using the log-rank statistic. The primary and secondary end points of mortality were analyzed using the Cox proportional hazards regression model, in which variables with clinical and pathophysiological relevance plus univariate *P* < .05 were chosen for multivariable analyses; aortic valve surgery was analyzed as a time-dependent variable. Penalized smoothing splines were used to illustrate the risk of mortality over the range of the LV parameters. A landmark analysis^19^ was used to assess whether early aortic valve surgery was associated with better survival. After 6 months, surviving patients were further divided into 2 groups—those with and without aortic valve surgery within 6 months. Then, the results were plotted according to 3 LVESDi strata (<20, 20 to <25, and ≥25 mm/m^2^). To evaluate survival differences between Japanese and Taiwanese patients, the inverse probability of treatment weighting method was used to control confounding as previously described.^20^ All statistical analyses were performed using JMP, version 16 (JMP Statistical Discovery LLC); SAS, version 9.4 (SAS Institute); and R, version 4.1.2 (R Project for Statistical Computing). Two-sided *P* < .05 was considered statistically significant.

## Results

### Baseline Characteristics

The final study cohort included 1259 patients with chronic, moderately severe to severe AR (mean [SD] age, 64 [17] years; 325 [26%] female; 934 [74%] male), of whom 515 (41%) were Japanese and 744 (59%) were Taiwanese (Table 1). The mean (SD) body surface area was 1.67 (0.21) m^2^; LVEF, 55% (11%); LVESDi, 24.7 (5.7) mm/m^2^; LVESVi, 50.1 (28.0) mL/m^2^; and indexed mid–ascending aorta size, 24.7 (5.5) mm/m^2^. As compared with Taiwanese patients, Japanese patients had similar prevalence of bicuspid aortic valves, were less symptomatic, and had a smaller body surface area, lower CCI score, greater proportion of female patients, greater proportion who had undergone aortic valve surgery, larger LV size (both absolute and indexed LV dimensions and volumes), lower LVEF (mean [SD], 52% [11%] vs 58% [10%]), lower LV filling pressure, smaller AR vena contracta, and greater pressure half time (Table 1). In this cohort, LV volumes were derived from biplane disk summation in 1107 patients (89%) and single plane in 140 (11%).

**Table 1.** Baseline Characteristics

Characteristic	Patients			*P* value
	Total (N = 1259)	Taiwan (n = 744)	Japan (n = 515)	
Age, mean (SD), y	64 (17)	64 (17)	65 (17)	.20
Sex, No. (%)				
Female	325 (26)	169 (23)	156 (30)	.002
Male	934 (74)	575 (77)	359 (70)	
Body surface area, mean (SD), m^2^	1.67 (0.21)	1.70 (0.20)	1.61 (0.21)	<.001
BMI, mean (SD)	23.0 (4.0)	23.6 (3.9)	22.3 (4.1)	<.001
Blood pressure, mean (SD), mm Hg				
Systolic	135 (20)	135 (18)	136 (22)	.49
Diastolic	65 (13)	66 (12)	64 (13)	.001
Pulse pressure, mean (SD), mm Hg	70 (20)	69 (20)	72 (20)	.006
Heart rate, mean (SD), bpm	70 (13)	71 (13)	67 (13)	<.001
Hypertension, No. (%)	784 (63)	453 (61)	331 (64)	.28
Hyperlipidemia, No. (%)	273 (22)	131 (18)	142 (28)	<.001
Diabetes, No. (%)	102 (8)	58 (8)	44 (9)	.65
Connective tissue disease, No. (%)	95 (8)	47 (6)	48 (9)	.053
Coronary artery disease, No. (%)	203 (16)	175 (24)	28 (5)	<.001
Charlson Comorbidity Index score, No. (IQR)	1 (0-2)	1 (0-2)	0 (0-2)	<.001
NYHA functional class, No./total No. (%)				
I^a^	700/1245 (56)	381/731 (52)	319/514 (62)	.002
II	418/1245 (34)	267/731 (37)	151/514 (29)	
III and IV	127/1245 (10)	83/731 (11)	44/514 (9)	
BAV, No./total No. (%)	243/1259 (19)	150/744 (20)	93/515 (18)	.35
BAV fusion type, No./total No. (%)^b^				
RL	177/242 (73)	108/149 (72)	69/93 (74)	.31
RN	50/242 (21)	34/149 (23)	16/93 (17)	
LN	15/242 (6)	7/149 (5)	8/93 (9)	
LVEF via Simpson method, mean (SD), %	55 (11)	58 (10)	52 (11)	<.001
LVEDD, mean (SD), mm	60 (8)	60 (7)	61 (8)	.001
LVEDDi, mean (SD), mm/m^2^	36.6 (5.2)	35.4 (4.7)	38.2 (5.4)	<.001
LVESD, mean (SD), mm	41 (9)	39 (8)	43 (9)	<.001
LVESDi, mean (SD), mm/m^2^	24.7 (5.7)	22.9 (4.8)	27.0 (5.9)	<.001
LVESDi >25 mm/m^2^, No. (%)	481 (38)	186 (25)	295 (57)	<.001
LVEDVi, mean (SD), mL/m^2^	108.1 (39.3)	99.3 (36.9)	120.5 (39.3)	<.001
LVESVi, mean (SD), mL/m^2^	50.1 (28.0)	43.4 (23.8)	59.4 (30.8)	<.001
LAVi, mean (SD), mL/m^2^	38.0 (17.3)	31.3 (12.7)	46.4 (18.9)	<.001
E/e′, mean (SD)	13 (6)	14 (6)	12 (6)	<.001
RVSP, mean (SD), mm Hg	30 (10)	31 (10)	30 (9)	.06
Atrial fibrillation, No. (%)	96 (8)	52 (7)	44 (9)	.31
AR vena contracta, mean (SD), mm	6.6 (1.7)	7.0 (1.9)	6.1 (1.3)	<.001
AR EROA, mean (SD), cm^2^	0.28 (0.10)	NA	0.28 (0.10)	NA
AR regurgitant volume, mean (SD), mL	61 (17)	NA	61 (17)	NA
AR pressure half time, mean (SD), msec	380 (104)	349 (110)	387 (101)	.001
Aorta dimensions, mean (SD)				
Annulus diameter, mm	23.4 (3.2)	23.4 (3.3)	23.4 (2.8)	.76
Indexed annulus, mm/m^2^	14.2 (1.9)	13.9 (1.9)	14.6 (1.9)	<.001
Sinus of valsalva diameter, mm	40.5 (8.2)	41.6 (8.4)	39.0 (7.7)	<.001
Indexed sinus of valsalva, mm/m^2^	24.4 (4.9)	24.5 (4.9)	24.3 (4.8)	.50
Mid–ascending aorta diameter, mm	40.4 (8.1)	44.2 (8.1)	37.7 (7.1)	<.001
Indexed mid–ascending aorta, mm/m^2^	24.7 (5.5)	26.1 (5.3)	23.7 (5.3)	<.001
Surgery parameters				
AV surgery, No. (%)	483 (38)	258 (35)	225 (44)	.001
Surgery and surgical indications, No./total No. (%)^a^				
Symptoms	285/481 (59)	198/258 (77)	87/223 (39)	<.001
LVEF <50%	47/481 (10)	2/258 (<1)	45/223 (20)	<.001
LVESDi >50 mm or 25 mm/m^2^	75/481 (16)	24/258 (9)	51/223 (23)	<.001
Aortic aneurysm	21/481 (4)	14/258 (5)	7/223 (3)	<.001
LVEDD >65 mm	36/481 (7)	10/258 (4)	26/223 (12)	<.001
Early surgery	17/481 (4)	10/258 (4)	7/223 (3)	<.001
AV repair	24/483 (5)	8/258 (3)	16/225 (7)	.04
Bioprosthesis	330/457 (72)	167/249 (67)	163/208 (78)	.006
Concomitant aorta surgery^c^	170/481 (35)	115/258 (45)	55/223 (25)	<.001
Concomitant CABG	48/483 (10)	29/258 (11)	19/223 (9)	.31

Abbreviations: AR, aortic regurgitation; AV, aortic valve; BAV, bicuspid aortic valve; BMI, body mass index (calculated as weight in kilograms divided by height in meters squared); bpm, beats per minute; CABG, coronary artery bypass grafting; E/e′, peak mitral inflow velocity to early diastolic mitral annular velocity ratio; EROA, effective regurgitant orifice area; LAVi, left atrial volume index; LN, left noncoronary cusp fusion; LVEDD, left ventricular end-diastolic dimension; LVEDDi, left ventricular end-diastolic dimension index; LVEDVi, left ventricular end-diastolic volume index; LVEF, left ventricular ejection fraction; LVESD, left ventricular end-systolic dimension; LVESDi, left ventricular end-systolic dimension index; LVESVi, left ventricular end-systolic volume index; NA, not applicable; NYHA, New York Heart Association; RL, right-left coronary cusp fusion; RN, right noncoronary cusp fusion; RVSP, right ventricular systolic pressure.

^a^ Two patients received surgery elsewhere and had unknown surgical indications; thus, they were excluded from analysis.

^b^ Excluding 1 patient with unknown phenotype.

^c^ Excluding 2 patients.

### Surgical Procedures and Reasons for Surgery

**Figure 1.**
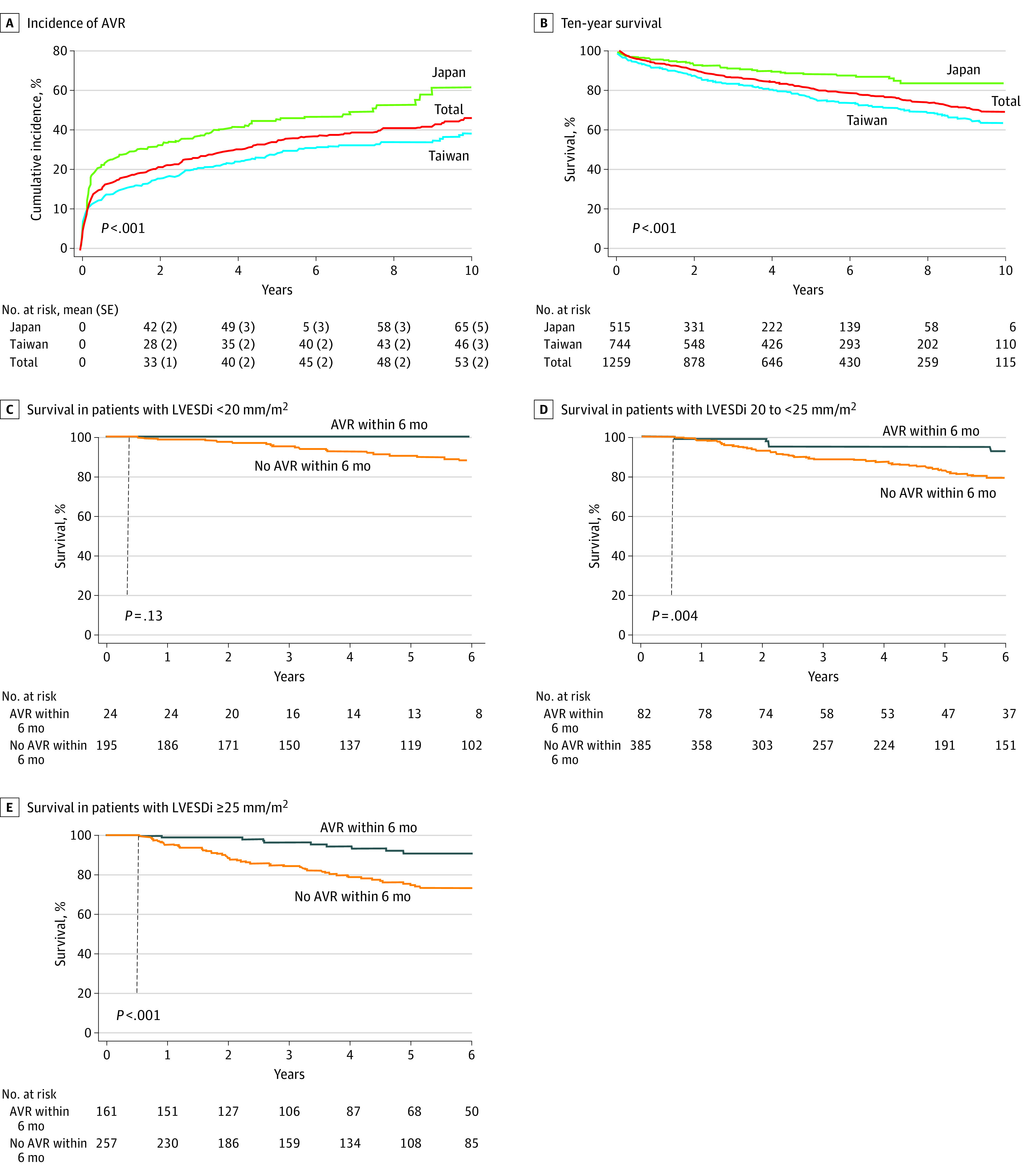
Incidence of Aortic Valve Repair (AVR), Overall Survival, and Survival Based on Left Ventricular End-Systolic Diameter Index (LVESDi) Strata

The distribution of surgical procedures is shown in Table 1. At a median follow-up of 4.1 years (IQR, 1.56-7.24 years), 483 patients (38%) underwent aortic valve surgery; of those, 170 (35%) had concomitant aorta surgery and 48 (10%) had concomitant coronary artery bypass surgery. Six of 483 patients (1%) died within 30 days after aortic valve surgery. The mean (SD) 8- and 10- year aortic valve surgery incidence in the total cohort was 48% (2%) and 53% (2%), respectively (Figure 1A). Compared with Taiwanese patients, Japanese patients had more aortic valve surgeries (mean [SD] 10-year incidence, 65% [5%] vs 46% [3%]; *P* < .001) (Figure 1A) but less concomitant aorta surgeries, reflected by smaller mid–ascending aorta sizes than in Taiwanese patients (Table 1); also, Japanese patients had higher rates of aortic valve repair and bioprosthesis use, and aortic valve surgery was more frequently performed owing to LV criteria rather than symptoms in Japanese patients (Table 1). However, all 3 LV parameters (LVEF, LVESDi, and LVESVi) were associated with aortic valve surgery (eTable 1 in Supplement 1). At the time of aortic valve surgery, Japanese patients had significantly larger LV end-diastolic dimension index (LVEDDi) and LVESDi values than Taiwanese patients (LVEDDi: mean [SD], 39 [5] mm/m^2^ vs 38 [5] mm/m^2^; LVESDi: mean [SD], 29 [5] mm/m^2^ vs 26 [5] mm/m^2^; both *P* < .001).

### Factors Associated With ACD and CVD

Median follow-up was 4.1 years (IQR, 1.5-7.2 years), with a maximum of 13 years, during which 240 patients (19%) died (201 deaths [84%] were during medical management; 39 [16%] were after aortic valve surgery). Results of univariate analysis are shown in eTables 2 and 3 in Supplement 1. In multivariate analysis in the total cohort, all 3 LV indexes (LVEF: HR per 10%, 0.80; 95% CI, 0.70-0.92; *P* = .002; LVESDi: HR, 1.04; 95% CI, 1.01-1.06; *P* < .002; LVESVi: HR per 10 mL/m^2^, 1.11; 95% CI, 1.05-1.17; *P* < .001) were independently associated with ACD during medical surveillance (Table 2). In Japanese patients, only LVEF was independently associated with ACD during medical surveillance (n = 38); the hazard ratios (HRs) for LVESDi and LVESVi were greater than 1, although the findings were not statistically significant, possibly due to low event count.

**Table 2.** Multivariate Cox Proportional Hazards Regression Analysis for Factors Associated With All-Cause Death During Medical Surveillance^a^

Factor	Total (N = 1259)		Taiwan (n = 744)		Japan (n = 515)	
	HR (95% CI)	*P* value	HR (95% CI)	*P* value	HR (95% CI)	*P* value
**LVEF**						
Age	1.06 (1.05-1.08)	<.001	1.06 (1.05-1.08)	<.001	1.06 (1.02-1.10)	<.001
Female	1.42 (1.04-1.94)	.02	1.45 (1.02-2.05)	.04	1.56 (0.80-3.06)	.19
Charlson Comorbidity Index	1.26 (1.18-1.35)	<.001	1.20 (1.11-1.30)	<.001	1.43 (1.25-1.64)	<.001
NYHA functional class						
I	1 [Reference]	NA	1 [Reference]	NA	1 [Reference]	NA
II	0.97 (0.69-1.36)	.88	1.10 (0.76-1.61)	.59	NA	NA
III and IV	1.67 (1.06-2.65)	.02	2.12 (1.26-3.57)	.004	NA	NA
LVEF per 10%	0.80 (0.70-0.92)	.002	0.82 (0.71-0.96)	.02	0.72 (0.55-0.97)	.03
Taiwanese vs Japanese	2.28 (1.57-3.32)	<.001	NA	NA	NA	NA
**LVESDi^b^**						
Age	1.06 (1.05-1.08)	<.001	1.06 (1.04-1.08)	<.001	1.06 (1.02-1.10)	.004
Female	1.32 (0.97-1.79)	.07	1.35 (0.95-1.91)	.08	1.36 (0.70-2.64)	.35
Charlson Comorbidity Index	1.28 (1.20-1.37)	<.001	1.21 (1.12-1.31)	<.001	1.51 (1.31-1.72)	<.001
NYHA functional class						
I	1 [Reference]	NA	1 [Reference]	NA	1 [Reference]	NA
II	0.99 (0.71-1.38)	.96	1.11 (0.77-1.61)	.55	NA	NA
III-IV	1.77 (1.14-2.76)	.01	2.17 (1.32-3.58)	.002	NA	NA
LVESDi, mm/m^2^	1.04 (1.01-1.06)	.002	1.04 (1.01-1.07)	.003	1.03 (0.98-1.08)	.17
Taiwanese vs Japanese	2.43 (1.65-3.56)	<.001	NA	NA	NA	NA
**LVESVi**						
Age	1.07 (1.05-1.08)	<.001	1.07 (1.05-1.08)	<.001	1.06 (1.02-1.10)	<.001
Female	1.53 (1.12-2.09)	.008	1.53 (1.07-2.19)	.02	1.59 (0.81-3.13)	.17
Charlson Comorbidity Index	1.28 (1.20-1.37)	<.001	1.22 (1.13-1.33)	<.001	1.48 (1.29-1.69)	<.001
NYHA functional class						
I	1 [Reference]	NA	1 [Reference]	NA	1 [Reference]	NA
II	0.95 (0.67-1.33)	.77	1.05 (0.72-1.54)	.77	NA	NA
III and IV	1.67 (1.06-2.61)	.02	2.12 (1.27-3.52)	.003	NA	NA
LVESVi per 10 mL/m^2^	1.11 (1.05-1.17)	<.001	1.11 (1.04-1.19)	.002	1.10 (1.00-1.20)	.05
Taiwanese vs Japanese	2.51 (1.70-3.70)	<.001	NA	NA	NA	NA

Abbreviations: HR, hazard ratio; LVEF, left ventricular ejection fraction; LVESDi, left ventricular end-systolic diameter index; LVESVi, left ventricular end-systolic volume index; NA, not applicable; NYHA, New York Heart Association.

^a^ All-cause death occurred in 163 Taiwanese and 38 Japanese patients.

^b^ After adjustment for age, sex, Charlson Comorbidity Index score, and NYHA functional status, the HR per 1 mm for LVESD was 1.00 (95% CI, 0.98-1.02; *P* = .45) in the entire cohort, 1.02 (95% CI, 1.00-1.04; *P* = .04) in the Taiwan cohort, and 1.01 (95% CI, 0.97-1.05; *P* = .39) in the Japan cohort.

During total follow-up in Taiwanese patients, all 3 LV indexes (LVEF, LVESDi, and LVESVi) were independently associated with ACD (n = 192) (Table 3). In Japanese patients, there was no association of LVEF, LVESDi, and LVESVi with ACD (n = 48) (Table 3).

**Table 3.** Multivariate Cox Proportional Hazards Regression Analysis for Factors Associated With All-Cause Death During Total Follow-up^a^

Factor	Total (N = 1259)		Taiwan (n = 744)		Japan (n = 515)	
	HR (95% CI)	*P* value	HR (95% CI)	*P* value	HR (95% CI)	*P* value
**LVEF**						
Age	1.06 (1.05-1.08)	<.001	1.06 (1.05-1.08)	<.001	1.06 (1.03-1.09)	<.001
Female	1.42 (1.06-1.89)	.02	1.55 (1.12-2.15)	.008	NA	NA
Charlson Comorbidity Index	1.28 (1.20-1.37)	<.001	1.24 (1.16-1.34)	<.001	1.40 (1.23-1.59)	<.001
NYHA functional class						
I	1 [Reference]	NA	1 [Reference]	NA	1 [Reference]	NA
II	1.02 (0.75-1.39)	.90	1.18 (0.83-1.67)	.40	0.69 (0.34-1.42)	.30
III and IV	1.66 (1.09-2.53)	.02	1.80 (1.10-2.92)	.01	1.76 (0.77-4.00)	.20
LVEF per 10%	0.80 (0.70-0.90)	<.001	0.81 (0.70-0.93)	.002	0.80 (0.62-1.04)	.09
Time-dependent AVS	0.58 (0.39-0.85)	.005	0.56 (0.36-0.88)	.01	0.67 (0.31-1.43)	.30
Taiwanese vs Japanese	2.29 (1.63-3.21)	<.001	NA	NA	NA	NA
**LVESDi**						
Age	1.06 (1.05-1.08)	<.001	1.06 (1.05-1.08)	<.001	1.06 (1.03-1.09)	<.001
Female	1.34 (1.01-1.77)	.04	1.47 (1.07-2.02)	.01	NA	NA
Charlson Comorbidity Index	1.30 (1.22-1.38)	<.001	1.26 (1.17-1.35)	<.001	1.44 (1.27-1.63)	<.001
NYHA functional class						
I	1 [Reference]	NA	1 [Reference]	NA	1 [Reference]	NA
II	1.07 (0.79-1.46)	.65	1.24 (0.88-1.74)	.20	0.73 (0.35-1.51)	.40
III and IV	1.80 (1.21-2.69)	.004	1.91 (1.20-3.03)	.006	2.06 (0.89-4.78)	.09
LVESDi, mm/m^2^	1.04 (1.01-1.06)	.001	1.04 (1.02-1.07)	.001	1.02 (0.97-1.07)	.50
Time-dependent AVS	0.56 (0.39-0.83)	.003	0.53 (0.34-0.83)	.005	0.68 (0.31-1.46)	.30
Taiwanese vs Japanese	2.38 (1.69-3.36)	<.001	NA	NA	NA	NA
**LVESVi**						
Age	1.07 (1.05-1.08)	<.001	1.07 (1.05-1.08)	<.001	1.06 (1.03-1.09)	<.001
Female	1.47 (1.10-1.97)	.01	1.59 (1.14-2.22)	.006	NA	NA
Charlson Comorbidity Index	1.30 (1.23-1.39)	<.001	1.27 (1.18-1.36)	<.001	1.42 (1.25-1.61)	<.001
NYHA functional class						
I	1 [Reference]	NA	1 [Reference]	NA	1 [Reference]	NA
II	1.04 (0.76-1.42)	.81	1.20 (0.84-1.70)	.30	0.73 (0.36-1.49)	.40
III and IV	1.75 (1.16-2.64)	.008	1.91 (1.19-3.08)	.007	1.83 (0.78-4.31)	.20
LVESVi per 10 mL/m^2^	1.08 (1.03-1.13)	.001	1.07 (1.02-1.14)	.01	1.06 (0.97-1.16)	.20
Time-dependent AVS	0.54 (0.37-0.81)	.002	0.52 (0.33-0.83)	.006	0.64 (0.30-1.39)	.30
Taiwanese vs Japanese	2.28 (1.62-3.22)	<.001	NA	NA	NA	NA

Abbreviations: AVS, aortic valve surgery; HR, hazard ratio; LVEF, left ventricular ejection fraction; LVESDi, left ventricular end-systolic diameter index; LVESVi, left ventricular end-systolic volume index; NA, not applicable; NYHA, New York Heart Association.

^a^ All-cause death occurred in 192 Taiwanese and 48 Japanese patients.

Multivariable factors associated with CVD (n = 74) are shown in eTables 4 and 5 in Supplement 1. In the total cohort, all 3 LV indexes (LVEF: HR per 10%, 0.69; 95% CI, 0.56-0.85; *P* < .001; LVESDi: HR, 1.05; 95% CI, 1.01-1.09; *P* = .01; LVESVi per 10 mL/m^2^: HR, 1.15; 95% CI, 1.06-1.24; *P* < .001) were associated with CVD during medical surveillance (eTable 4 in Supplement 1). During total follow-up, LVEF and LVESDi were independently associated with CVD in Taiwanese patients; greater LVESVi was not associated with CVD (eTable 5 in Supplement 1). In Japanese patients, all 3 LV indexes were independently associated with CVD after adjusting for age (eTable 5 in Supplement 1).

### Survival

Mean (SD) 8-year survival in the entire cohort was 74% (2%); in Japanese patients, 84% (3%); and in Taiwanese patients, 69% (2%). Mean (SD) 10-year survival in the entire cohort was 69% (2%); in Japanese patients, 84% (3%); and in Taiwanese patients, 64% (2%) (*P* < .001 for Japanese vs Taiwanese patients). Taiwanese patients had greater risk of death, both with age and sex adjustment (HR, 2.19; 95% CI, 1.59-3.02) and without (HR, 2.45; 95% CI, 1.78-3.38) (both *P* < .001) (Figure 1B). The inverse probability of treatment weighting analysis matching for 10 covariates showed that Japanese patients had better survival than Taiwanese patients (HR, 2.23; 95% CI, 1.51-3.30; *P* < .001) (eFigure 2 in Supplement 1).

### Association Between Aortic Valve Surgery and Survival

In univariable analysis, aortic valve surgery was associated with reduced ACD in the entire cohort and in the Taiwanese and Japanese cohorts (eTable 3 in Supplement 1). In multivariable analysis, aortic valve surgery was associated with reduced ACD in the entire cohort and in the Taiwanese cohort; for the Japanese cohort, ACD was not associated with aortic valve surgery, possibly because of a small mortality event count (Table 3). For CVD, aortic valve surgery was associated with better survival in univariable analysis in the entire cohort (HR, 0.59; 95% CI, 0.35-0.97; *P* = .03). In multivariable analysis, aortic valve surgery was not associated with CVD in the entire cohort (eTable 5 in Supplement 1). In 3 strata of LVESDi (<20, 20 to <25, and ≥25 mm/m^2^) in the entire cohort, patients who underwent aortic valve surgery within 6 months had better survival than those who did not undergo aortic valve surgery within 6 months (776 [80%] had never undergone aortic valve surgery at the end of follow-up) (Figure 1C). Aortic valve surgery within 6 months was also associated with improved survival in subgroups with LVESVi less than 46 mL/m^2^ or 46 mL/m^2^ or greater.

### LV Cutoffs During Total Follow-up for ACD and CVD

**Figure 2.**
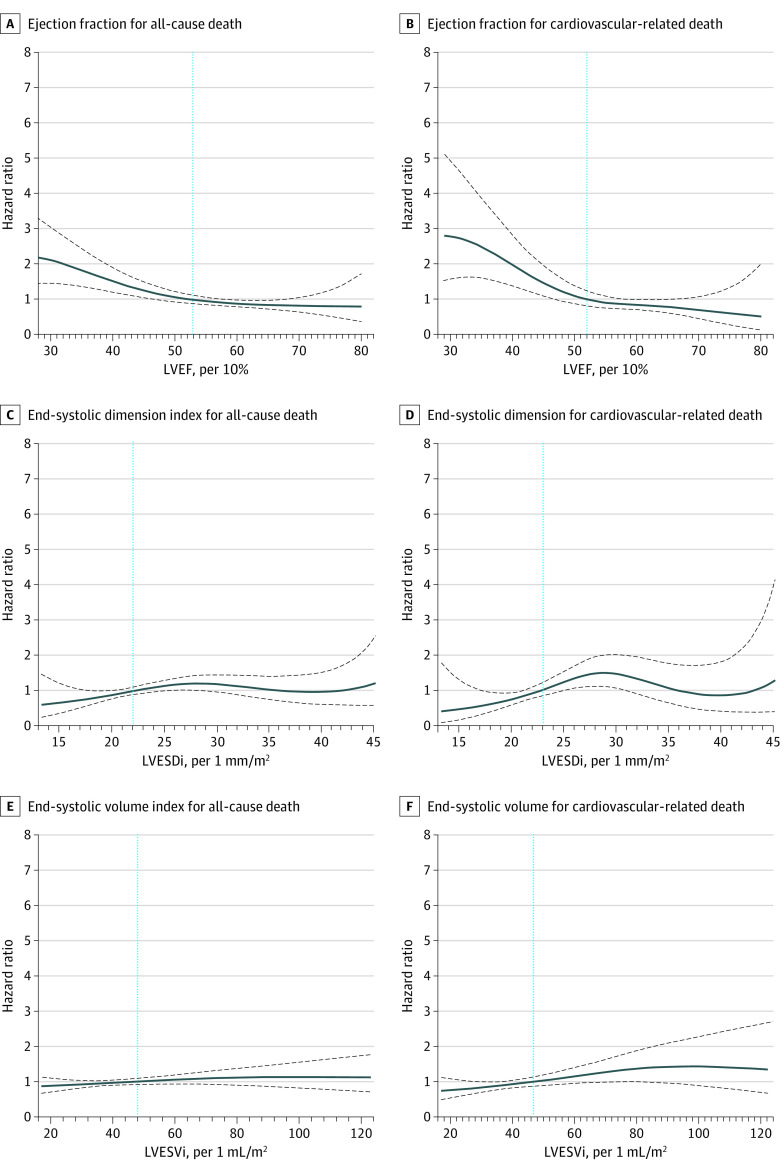
Adjusted Cutoffs for Risk of Death in the Entire Asian Cohort Findings were similar to the Western population.^9,12,21^ Solid lines represent mean values, and dashed lines represent 95% CIs. LVEF indicates left ventricular ejection fraction; LVESDi, left ventricular end-systolic dimension index; and LVESVi, left ventricular end-systolic volume index.

In the entire cohort, age- and sex-adjusted cutoffs associated with increased risk for ACD were 53% for LVEF, 22 mm/m^2^ for LVESDi, and 48 mL/m^2^ for LVESVi. The corresponding cutoffs for CVD were 52%, 23 mm/m^2^, and 46 mL/m^2^, respectively (Figure 2).

### Subgroups of Patients With LVEF of 50% or Greater and NYHA Class I

In asymptomatic patients with an LVEF of 50% or greater (n = 573; 63 deaths), LVESDi and LVESVi were associated with ACD during medical surveillance. However, LVEF was not (eTable 6 in Supplement 1).

### Comparison Between Asian and Western Individuals With Significant AR

Compared with Western individuals from a prior study by some of us^22^ (eTable 7 in Supplement 1), Asian individuals consisted of more females and had smaller body surface area, higher CCI scores, more right-noncoronary cusp fusion, larger indexed LV dimensions or volumes, larger indexed aorta size, slightly lower 8-year survival, and lower incidence of aortic valve surgery yet similar postsurgery survival.

## Discussion

To our knowledge, this is the only multicenter study of an Asian cohort with hemodynamically significant AR. We also report, for the first-time to our knowledge, cutoffs of LVEF, LVESDi, and LVESVi for comparison with cutoff values for Western individuals.

In this study, first, the Japanese cohort was composed of more women, was more asymptomatic, and had lower CCI scores, smaller body surface area, larger LV, more aortic valve surgeries, smaller aorta size, and less concomitant aorta surgery. Second, in the entire cohort, age- and sex-adjusted cutoffs for ACD were 53% for LVEF, 22 mm/m^2^ for LVESDi, and 48 mL/m^2^ for LVESVi, and the corresponding cutoffs for CVD were 52%, 23 mm/m^2^, and 46 mL/m^2^, respectively (Figure 2), cutoffs similar to those for the Western population. Third, in Taiwanese individuals, LVEF, LVESDi, and LVESVi were independently associated with ACD and CVD, whereas in Japanese individuals, all 3 LV parameters were associated with CVD, yet none were associated with ACD. Fourth, aortic valve surgery was associated with ACD and CVD in the entire cohort in univariable analysis and in 3 strata of LVESDi subgroups and 2 strata of LVESVi subgroups. Fifth, compared with Western individuals, Asian individuals had larger indexed LV size, slightly lower 8-year survival, and lower incidence of aortic valve surgery yet similar postsurgical survival (eTable 7 in Supplement 1).

In patients with AR, the most recent, 2020-2021 US and European guidelines^23,24^ updated the timing for surgical intervention (eg, LVEF ≤55% and LVESD >20 mm/m^2^) compared with prior versions (eg, LVEF <50% and LVESDi >25 mm/m^2^).^3^ These guideline changes were based on recent studies conducted in Western individuals. So far, no evidence has shown that these results hold true in Asian individuals, who compose 60% of the global population but for whom data on AR are scarce. A recent study by some of us^12^ and the current study (eTable 7 in Supplement 1) demonstrated interethnic differences in AR clinical presentation. Notably, Asian individuals had larger indexed LV size and fewer aortic valve surgeries, which may have been associated with slightly lower 8-year survival than that among Western individuals. Despite interethnic differences, cutoffs for LVEF and LVESDi seemed similar compared with Western individuals except for a slightly higher LVESVi cutoff (Figure 2). In our study, risk of death started to rise before LVEF decreased below 50% (≤53% for ACD and ≤52% for CVD). This finding resonated with recommendations from the most recent Western guidelines.^10,21,23,24^ Thus, contemporary data from both East and West suggest that an LVEF of 50% to 55% is a reasonable cutoff for timely surgery referral. Most importantly, the LVEF of the current study was derived from the biplane Simpson method,^16^ which is more accurate and is identical with the LVEF calculation method used by de Meester et al.^10^

For both ACD and CVD, the LVESDi cutoff in this study (22-23 mm/m^2^) (Figure 2) was similar to cutoffs derived from Western individuals, suggesting a similar timing of surgical referral.^9,10^ Yet, the aortic valve repair rate was considerably lower in Asian vs US patients^12,25^; hence, clinicians should weigh the benefits of surgery against complications of prosthetic valve implantation in the long term. Also, the use of LVESDi was paramount to evaluate the degree of AR-related LV dilatation, especially in Asian individuals who had similar absolute LV size yet smaller stature compared with Western individuals.^9^ For LVESVi, the cutoff in Asian individuals (46-48 mL/m^2^) (Figure 2) was slightly higher than cutoffs derived from Western individuals (>40-45 mL/m^2^),^21^ possibly due to intercountry variations of indexed LV volumes^26^ and because Asian individuals were at a later stage of AR natural history (eTable 7 in Supplement 1).

Compared with Taiwanese individuals, Japanese individuals had better survival despite larger indexed LV size (Figure 1B and eFigure 2 in Supplement 1), suggesting that Japanese individuals, who have the longest life expectancy in the world,^27^ may be less vulnerable to AR-related LV remodeling. These observations could potentially be explained by earlier surgery at asymptomatic status, higher aortic valve surgery incidence, uncollected confounders (ie, operative skill, medical compliance), diet, culture, low prevalence of obesity, and educational level.^28^

Interestingly, both Japanese and Taiwanese individuals had larger LVESDi at the time of aortic valve surgery (ie, late referral) than the US cohort (mean [SD], 21 [4] mm/m^2^),^22^ yet the mean (SD) 8-year post-aortic valve surgery survival was similar (85% [3%] in Asian individuals in this study vs 84% [2%] in Western individuals^22^) (eTable 7 in Supplement 1). This finding suggests that despite interethnic differences, there are benefits to aortic valve surgery.

### Clinical Implications

Findings from this multicenter Asian study have potential clinical implications. First, our observed cutoffs of 52% to 53% for LVEF, 22 to 23 mm/m^2^ for LVESDi, and 46 to 48 mL/m^2^ for LVESVi help fill the knowledge gap of lacking Asian data in recent Western guidelines.^23,24^ It seemed reasonable for Asian individuals to abide by Western guidelines in the treatment of AR. With a total patient number of almost 4000 from previous studies^8,9,10,21^ and this study, our findings help generalize the results from prior Western studies. Second, this study highlights the importance of using indexed LV parameters as a common communication language in patients with VHD. Third, despite late presentation (larger indexed LV size) and lower prevalence of aortic valve surgery in Asian individuals, the postsurgical survival was similar to that among Western individuals (eTable 7 in Supplement 1), suggesting that earlier surgery and a more proactive approach in treating Asian patients with AR is associated with better overall survival. Finally, although LVESVi was not yet integrated into current guidelines for management of AR due to insufficient evidence,^24^ our results plus those from recent studies^21,29^ provide added value and may serve as a risk-stratification tool for clinicians.

### Limitations

This study has limitations. It was a retrospective observational study from tertiary referral centers with the potential for selection bias. However, both Japan and Taiwan had compulsory health insurance^28^ with good access and equal opportunities to receive health care, including echocardiography, which may enhance the generalization of the results. Data from Taiwan did not have PISA quantification for AR, which may raise doubt regarding the accuracy of assessment of AR severity. Yet, 1 of us (L.T.Y.) who is experienced in AR assessment with relevant publications^9,21,22,30^ reviewed all echocardiograms de novo to discern AR severity. Also, compared with a US cohort,^22^ the indexed LV dimensions from Taiwanese individuals were larger and diastolic blood pressure and absolute LV size were similar, supporting the presence of hemodynamically significant AR. Given the relatively low mortality among Japanese individuals, there was insufficient power for finding LV cutoffs for death and for multivariable analysis, especially for CVD. Finally, prospective studies are needed to confirm benefits of early surgical timing using the LV cutoffs found herein. Future studies on usefulness of cardiac magnetic resonance imaging and global longitudinal strain in Asian patients with AR are also of interest.

## Conclusions

In this large, multicenter, contemporary cohort study of Asian patients with hemodynamically significant AR, the cutoffs of LVEF, LVESDi, and LVESVi values associated with increased risks of death were similar to what have been reported for the Western population. Asian individuals were at a later stage of AR natural history with a larger indexed LV and lower rates of aortic valve surgery yet had similar postsurgical survival compared with Western individuals. These findings suggest that current guidelines seem applicable to Asian individuals with AR and that earlier surgical timing using indexed LV size as guidance may be associated with improvements in overall outcomes.

## Notes

Supplement 1. **eFigure 1.** Study Flow **eTable 1.** Factors Linked to Aortic Valve Surgical Intervention in Taiwanese and Japanese Individuals **eTable 2.** Univariate Cox Proportional Hazards Regression Analysis for Factors Associated With All-cause Death Under Medical Surveillance **eTable 3.** Univariate Cox Proportional Hazards Regression Analysis for Factors Associated With All-cause Death During Total Follow-Up **eTable 4.** Multivariate Cox Proportional Hazards Regression Analysis for Factors Associated With Cardiovascular Death Under Medical Surveillance **eTable 5.** Multivariate Cox Proportional Hazards Regression Analysis for Factors Associated With Cardiovascular Death During Total Follow-Up **eFigure 2.** Survival Between Japanese and Taiwanese Individuals Using Inverse Probability of Treatment Weighting Model **eTable 6.** Factors Associated With All-cause Death Under Medical Surveillance in Patients with LVEF of 50% or Greater and NYHA Class I **eTable 7.** Comparison Between Asian and Western Individuals

Supplement 2.
Data Sharing Statement
