# Supplementary material for: Analysis of Left Ventricular Indexes and Mortality Among Asian Adults With Hemodynamically Significant Chronic Aortic Regurgitation
Source: JAMA Netw Open. 2023 Mar 24;6(3):e234632. doi: 10.1001/jamanetworkopen.2023.4632 (PMC10313151; doi:10.1001/jamanetworkopen.2023.4632)
Supplement: Supplement 1. — eFigure 1. Study Flow eTable 1. Factors Linked to Aortic Valve Surgical Intervention in Taiwanese and Japanese Individuals eTable 2. Univariate Cox Proportional Hazards Regression Analysis for Factors Associated With All-cause Death Under Medical Surveillance eTable 3. Univariate Cox Proportional Hazards Regression Analysis for Factors Associated With All-cause Death During Total Follow-Up eTable 4. Multivariate Cox Proportional Hazards Regression Analysis for Factors Associated With Cardiovascular Death Under Medical Surveillance eTable 5. Multivariate Cox Proportional Hazards Regression Analysis for Factors Associated With Cardiovascular Death During Total Follow-Up eFigure 2. Survival Between Japanese and Taiwanese Individuals Using Inverse Probability of Treatment Weighting Model eTable 6. Factors Associated With All-cause Death Under Medical Surveillance in Patients with LVEF of 50% or Greater and NYHA Class I eTable 7. Comparison Between Asian and Western Individuals [file jamanetwopen-e234632-s001.pdf]

Supplementary Online Content

Yang LT, Lee CC, Su CH, et al. Analysis of left ventricular indexes and mortality among Asian adults with hemodynamically significant chronic aortic regurgitation. *JAMA Netw Open*. 2023;6(3):e234632. doi:10.1001/jamanetworkopen.2023.4632

eFigure 1. Study Flow
eTable 1. Factors Linked to Aortic Valve Surgical Intervention in Taiwanese and Japanese Individuals
eTable 2. Univariate Cox Proportional Hazards Regression Analysis for Factors Associated With All-cause Death Under Medical Surveillance
eTable 3. Univariate Cox Proportional Hazards Regression Analysis for Factors Associated With All-cause Death During Total Follow-Up
eTable 4. Multivariate Cox Proportional Hazards Regression Analysis for Factors Associated With Cardiovascular Death Under Medical Surveillance
eTable 5. Multivariate Cox Proportional Hazards Regression Analysis for Factors Associated With Cardiovascular Death During Total Follow-Up
eFigure 2. Survival Between Japanese and Taiwanese Individuals Using Inverse Probability of Treatment Weighting Model
eTable 6. Factors Associated With All-cause Death Under Medical Surveillance in Patients with LVEF of 50% or Greater and NYHA Class I
eTable 7. Comparison Between Asian and Western Individuals

This supplementary material has been provided by the authors to give readers additional information about their work.

© 2023 Yang LT et al. *JAMA Network Open*.

eFigure 1. Study Flow

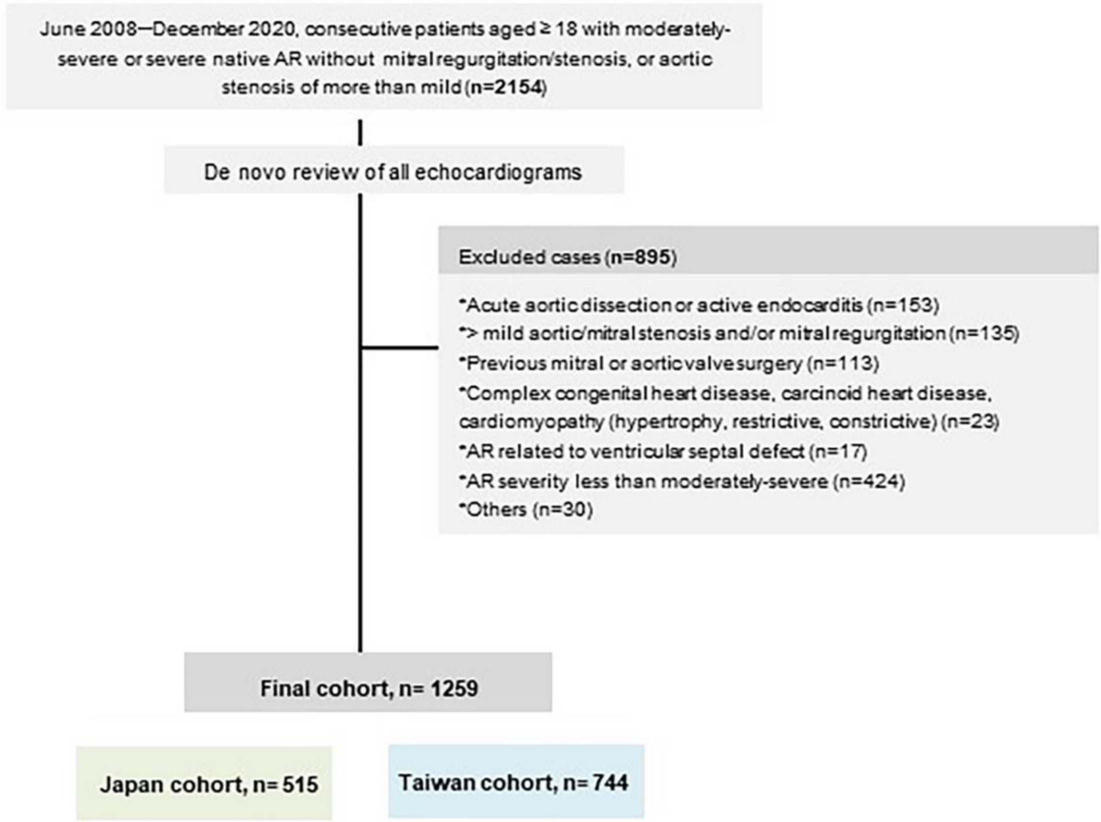

© 2023 Yang LT et al. *JAMA Network Open*.

eTable 1. Factors Linked to Aortic Valve Surgical Intervention in Taiwanese and Japanese Individuals

	**Taiwan (n=744, 258 AVS)**		**Japan (n=515, 225 AVS)**	
	HR(95% CI)	P value	HR(95% CI)	P value
Model 1*				
**LVESDi, mm/m^2**	1.06(1.04-1.08)	<0.0001	1.05(1.02-1.07)	<0.0001
Model 2*				
**LVEF per 10%**	0.84(0.74-0.96)	0.01	0.84(0.73-0.96)	0.01
Model 3*				
**LVESVi per 10ml/m^2**	1.12(1.08-1.17)	<0.0001	1.10(1.05-1.15)	<0.0001

See abbreviations from Table 2 and Table 3.

*All models were adjusted for age, sex, Charlson score, and New York Heart Association functional class.

© 2023 Yang LT et al. *JAMA Network Open*.

eTable 2. Univariate Cox Proportional Hazards Regression Analysis for Factors Associated With All-cause Death Under Medical Surveillance (n=201).

	**Total, n=1259**		**Taiwan, n=744**		**Japan, n=515**	
	**HR (95% CI)**	**P**	**HR (95% CI)**	**P**	**HR (95% CI)**	**P**
Age	1.08(1.06-1.09)	**<0.0001**	1.07(1.06-1.09)	**<0.0001**	1.08(1.05-1.12)	**<0.0001**
Female	1.55(1.15-2.07)	**0.004**	1.77(1.27-2.46)	**0.001**	1.51(0.79-2.86)	0.21
CCI	1.42(1.34-1.50)	**<0.0001**	1.36(1.28-1.45)	**<0.0001**	1.62(1.43-1.83)	**<0.0001**
NYHA (I as reference)		**<0.0001**		**<0.0001**		**0.01**
II	2.04(1.48-2.81)		2.09(1.46-2.98)		1.44(0.68-3.08)	
III/IV	6.8(4.70-9.93)		7.45(4.90-11.3)		4.52(1.91-10.7)	
LVEF per 10%	0.70(0.62-0.80)	**<0.0001**	0.66(0.56-0.78)	**<0.0001**	0.59(0.46-0.75)	**<0.0001**
LVESDi, mm/m^2	1.05(1.03-1.08)	**<0.0001**	1.08(1.05-1.11)	**<0.0001**	1.07(1.02-1.12)	**0.006**
LVESD, mm	1.00(0.98-1.02)	0.38	1.01(0.99-1.04)	0.08	1.01(0.97-1.05)	0.44
LVESDi <20 mm/m^2	Reference		Reference		Reference	
20-25 mm/m^2	1.31(0.90-1.92)	0.15	1.55(1.05-2.30)	**0.02**	1.01(0.22-4.63)	0.98
>25 mm/m^2	2.04(1.38-3.01)	**0.0002**	3.00(1.96-4.59)	**<0.0001**	2.23(0.53-9.44)	0.27
LVESVi per 10ml/ m^2*	1.06(1.00-1.12)	**0.02**	1.08(1.01-1.15)	**0.02**	1.14(1.05-1.24)	**0.006**
Taiwanese vs Japanese	1.99(1.39-2.84)	**<0.0001**				

CCI, Charlson comorbidity index. See Table 1 and 2 for abbreviations.

*After adjusting for age, the hazard ratio, 95%, and p value for LVESVi per 10ml/m^2 in the total cohort, Taiwanese and Japanese was 1.07(1.02-1.12; P=0.005), 1.10(1.04-1.17; P=0.001), and 1.13(1.03-1.22; p=0.009).

†After adjustment for age, sex, Charlson score, and NYHA, the HR, CI, and P value of LVESD in the entire- , Taiwan- , Japan-cohort was 1.00(0.98-1.02), P=0.45 ; 1.02(1.00-1.04), P=0.035; 1.01(0.97-1.05), P=0.39, respectively.

-

© 2023 Yang LT et al. *JAMA Network Open*.

eTable 3. Univariate Cox Proportional Hazards Regression Analysis for Factors Associated With All-cause Death During Total Follow-Up (n=240)

	**Total, n=1259**		**Taiwan, n=744**		**Japan, n=515**	
	**HR (95% CI)**	**P**	**HR (95% CI)**	**P**	**HR (95% CI)**	**P**
Age	1.07(1.06-1.09)	**<0.0001**	1.07(1.06-1.09)	**<0.0001**	1.08(1.05-1.11)	**<0.0001**
Female	1.54(1.17-2.02)	**0.002**	1.73(1.27-2.36)	**0.0007**	1.44(0.80-2.59)	0.22
CCI	1.47(1.39-1.54)	**<0.0001**	1.41(1.32-1.49)	**<0.0001**	1.63(1.45-1.83)	**<0.0001**
NYHA (I as reference)		**<0.0001**		**<0.0001**		**0.001**
II	1.85(1.38-2.48)		2.08(1.50-2.88)		1.00(0.50-2.01)	
III/IV	5.17(3.67-7.29)		5.31(3.58-7.88)		4.26(2.09-8.70)	
**Time-dependent AVS**	0.40(0.28-0.57)	**<0.001**	0.44(0.29-0.66)	**<0.001**	0.42(0.21-0.87)	**0.01**
LVEF per 10%	0.76(0.68-0.86)	**<0.0001**	0.70(0.61-0.81)	**<0.0001**	0.66(0.53-0.83)	**0.0007**
LVESDi, mm/m^2	1.03(1.01-1.05)	**0.003**	1.06(1.03-1.08)	**<0.0001**	1.04(1.00-1.09)	**0.03**
LVESD, mm	0.99(0.97-1.00)	0.21	1.00(0.98-1.02)	0.85	0.99(0.96-1.02)	0.89
LVESDi <20 mm/m^2	Reference		Reference		Reference	
20-25 mm/m^2	1.26(0.87-1.82)	0.20	1.50(1.02-2.20)	**0.03**	1.08(0.24-4.81)	0.91
>25 mm/m^2	1.64(1.14-2.37)	**0.007**	2.45(1.65-3.65)	**<0.0001**	1.82(0.43-7.61)	0.40
LVESVi per 10ml/ m^2*	1.00(0.95-1.05)	0.81	1.02(1.08-0.97)	0.45	1.07(0.98-1.14)	0.08
Taiwanese vs Japanese	2.19(1.59-3.02)	**<0.0001**				

CCI, Charlson comorbidity index. See Table 1 for abbreviations.

*After adjusting for age, the hazard ratio, 95%, and p value for LVESVi per 10ml/m^2 in the total cohort, Taiwanese and Japanese was 1.03(0.98-1.07; P=0.12), 1.05(1.00-1.10; P=0.042), and 1.11(1.01-1.19; p=0.017).

© 2023 Yang LT et al. *JAMA Network Open*.

eTable 4. Multivariate Cox Proportional Hazards Regression Analysis for Factors Associated With Cardiovascular Death Under Medical Surveillance (n=74)*

	Total, n=1259		Taiwan, n=744		Japan, n=515	
	**HR (95% CI)**	**P**	**HR (95% CI)**	**P**	**HR (95% CI)**	**P**
**Model 1: LVEF**						
Age	1.05(1.03-1.07)	**<0.0001**	1.05(1.03-1.07)	**<0.0001**		
Female	1.81(1.11-2.95)	**0.01**	1.55(0.89-2.69)	0.12		
Charlson comorbidity index	1.09(0.96-1.24)	0.15	0.97(0.82-1.13)	0.78		
NYHA (I as reference)						
II	1.15(0.65-2.02)	0.61	1.28(0.69-2.37)	0.42		
III/IV	2.56(1.21-5.41)	**0.01**	3.95(1.72-9.03)	**0.001**		
LVEF per 10%	0.69(0.56-0.85)	**0.0008**	0.72(0.57-0.91)	**0.007**	0.47(0.32-0.70)	**0.0006**
Taiwanese vs Japanese	3.34(1.75-6.39)	**<0.0001**				
**Model 2: LVESDi**						
Age	1.05(1.03-1.07)	**<0.0001**	1.05(1.02-1.07)	**<0.0001**		
Female	1.60(0.98-2.60)	0.06	1.41(0.81-2.44)	0.22		
Charlson comorbidity index	1.13(0.99-1.28)	0.05	1.00(0.86-1.17)	0.90		
NYHA (I as reference)						
II	1.22(0.70-2.14)	0.46	1.35(0.73-2.49)	0.33		
III/IV	2.88(1.41-5.89)	**0.003**	4.12(1.86-9.12)	**0.0005**		
LVESDi, mm/m^2	1.05(1.01-1.09)	**0.01**	1.05(1.00-1.10)	**0.04**	1.06(1.02-1.10)	**0.0009**
Taiwanese vs Japanese	3.33(1.70-6.49)	**0.0001**				
**Model 3: LVESVi**						
Age	1.05(1.03-1.08)	**<0.0001**	1.05(1.03-1.08)	**<0.0001**		
Female	2.00(1.21-3.29)	**0.007**	1.64(0.93-2.89)	0.08		
Charlson comorbidity index	1.13(1.00-1.28)	**0.04**	1.01(0.87-1.18)	0.80		
NYHA (I as reference)						
II	1.11(0.63-1.96)	0.69	1.25(0.67-2.34)	0.47		
III/IV	2.63(1.28-5.37)	**0.007**	4.04(1.81-8.99)	**0.0006**		
LVESVi per 10ml/m^2	1.15(1.06-1.24)	**0.0007**	1.12(1.02-1.24)	**0.02**	1.10(1.02-1.18)	**0.01**
Taiwanese vs Japanese	3.69(1.89-7.22)	**<0.0001**				

See Table 1 for abbreviations.

*Cardiovascular death occurred in 62 Taiwanese and 12 Japanese, respectively.

© 2023 Yang LT et al. *JAMA Network Open*.

eTable 5. Multivariate Cox Proportional Hazards Regression Analysis for Factors Associated With Cardiovascular Death During Total Follow-Up (n=94)*

	**Total, n=1259** 94 deaths		**Taiwan, n=744** 78 deaths		**Japan, n=515** 16 deaths	
	**HR (95% CI)**	**P**	**HR (95% CI)**	**P**	**HR (95% CI)**	**P**
**Model 1: LVEF**						
Age	1.05(1.03-1.06)	**<0.001**	1.05(1.03-1.07)	**<0.001**	1.06(1.01-1.11)	**0.01**
Female	1.93(1.25-2.99)	**0.003**	1.84(1.12-3.01)	**0.01**		
Charlson comorbidity index	1.15(1.02-1.28)	**0.02**	1.07(0.93-1.22)	0.3		
NYHA (I as reference)						
II	1.34(0.81-2.24)	0.25	1.49(0.85-2.60)	0.16		
III/IV	2.61(1.36-5.03)	**0.004**	3.07(1.47-6.42)	**0.003**		
LVEF per 10%	0.72 (0.60-0.87)	**<0.001**	0.74(0.61-0.92)	**0.005**	0.55(0.38-0.78)	**<0.001**
Time-dependent AVS	0.70(0.40-1.23)	0.22	0.68(0.36-1.27)	0.22		
Taiwanese vs Japanese	3.31(1.88-5.83)	**<0.001**				
**Model 2: LVESDi**						
Age	1.04(1.03-1.06)	**<0.001**	1.05(1.03-1.07)	**<0.001**	1.06(1.01-1.11)	**0.02**
Female	1.73(1.12-2.67)	**0.01**	1.66(1.02-2.71)	**0.04**		
Charlson comorbidity index	1.18(1.06-1.32)	**0.003**	1.09(0.96-1.25)	0.18		
NYHA (I as reference)						
II	1.42(0.86-2.36)	0.17	1.56(0.90-2.72)	0.12		
III/IV	2.93(1.56-5.53)	**<0.001**	3.32(1.63-6.77)	**<0.001**		
LVESDi, mm/m^2	1.05(1.01-1.09)	**0.008**	1.05(1.01-1.09)	**0.02**	1.07(1.00-1.15)	**0.04**
Time-dependent AVS	0.67(0.38-1.18)	0.17	0.63(0.33-1.19)	0.16		
Taiwanese vs Japanese	3.23(1.80-5.77)	**<0.001**				
**Model 3: LVESVi**						
Age	1.05(1.03-1.07)	**<0.001**	1.05(1.03-1.07)	**<0.001**	1.07(1.02-1.12)	**0.004**
Female	2.01(1.28-3.16)	**0.002**	1.86(1.12-3.07)	**0.02**		
Charlson comorbidity index	1.18(1.06-1.32)	**0.003**	1.10(0.96-1.25)	0.16		
NYHA (I as reference)						
II	1.38(0.83-2.30)	0.21	1.54(0.88-2.69)	0.13		
III/IV	2.82(1.48-5.36)	**0.002**	3.34(1.62-6.88)	**0.001**		
LVESVi per 10ml/m^2	1.10(1.03-1.18)	**0.004**	1.08(0.99-1.17)	0.06	1.18(1.06-1.30)	**0.002**
Time-dependent AVS	0.66(0.37-1.17)	0.15	0.65(0.34-1.25)	0.16		
Taiwanese vs Japanese	3.21(1.81-5.69)	**<0.001**				

During total follow-up, there were 78 and 16 cardiac deaths in Taiwanese and Japanese, respectively.
*Cardiovascular death occurred in 78 Taiwanese and 16 Japanese, respectively.

© 2023 Yang LT et al. *JAMA Network Open*.

eFigure 2. Survival between Japanese and Taiwanese individuals using inverse probability of treatment weighting (IPTW) model. Covariates related to survival were included in the model as the following: age, sex, body surface area, New York Heart Association functional class, Charlson comorbidity index, left ventricular ejection fraction, left ventricular end-systolic dimension index, left ventricular end-systolic volume index, left atrial volume index, and aortic valve surgery. Results showed that Japanese had better survival (hazard ratio, 2.23; 95% confidence interval, 1.51-3.30).

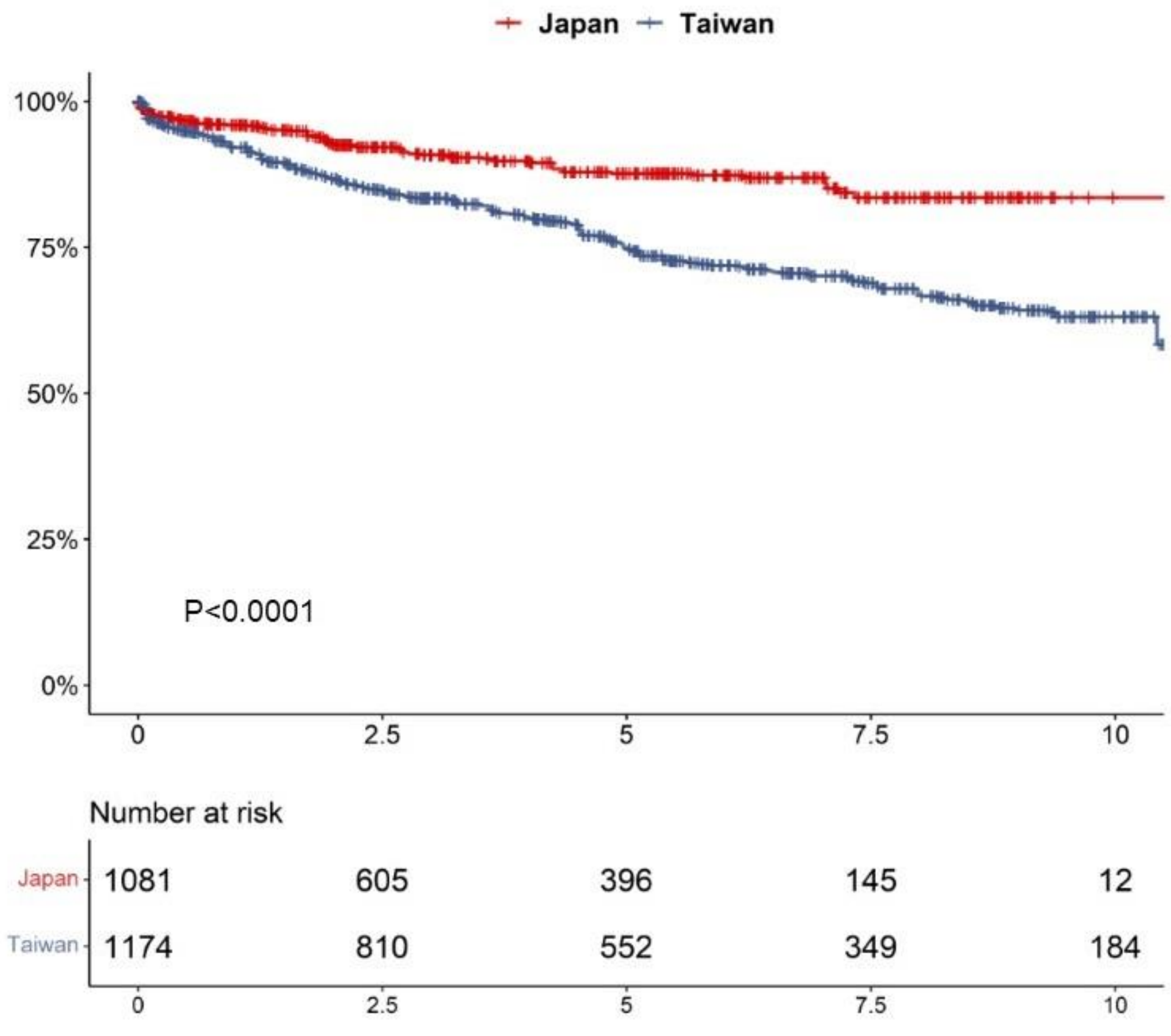

© 2023 Yang LT et al. *JAMA Network Open*.

eTable 6: Factors Associated With All-cause Death Under Medical Surveillance in Patients with LVEF of 50% or Greater and NYHA Class I*

	**Total, n=573**		**Taiwan, n=331**		**Japan, n=242**	
	**HR (95% CI)**	**P**	**HR (95% CI)**	**P**	**HR (95% CI)**	**P**
**Model 1: LVEF**						
Age	1.06(1.03-1.08)	**<0.0001**	1.05(1.03-1.08)	**<0.0001**	1.12(1.02-1.26)	**0.004**
Charlson comorbidity index	1.29(1.14-1.45)	**0.0002**	1.21(1.03-1.39)	**0.01**	1.57(1.23-1.98)	**0.0006**
Taiwanese vs Japanese	1.89(0.993.58)	**0.04**				
LVEF per 10%	0.88(0.58-1.32)	0.55	1.10(0.69-1.75)	0.66	0.35(0.07-1.19)	0.10
**Model 2: LVESDi**						
Age	1.06(1.03-1.09)	**<0.0001**	1.05(1.03-1.08)	**<0.0001**	1.14(1.03-1.29)	**0.002**
Charlson comorbidity index	1.27(1.12-1.43)	**0.0003**	1.19(1.02-1.36)	**0.02**	1.70(1.31-2.19)	**0.0002**
Taiwanese vs Japanese	2.49(1.294.80)	**0.003**				
LVESDi, mm/m^2	1.11(1.03-1.19)	**0.003**	1.10(1.01-1.19)	**0.02**	1.18(1.02-1.36)	**0.02**
**Model 3: LVESVi**						
Age	1.07(1.04-1.09)	**<0.0001**	1.06(1.03-1.09)	**<0.0001**	1.14(1.04-1.28)	**0.0007**
Charlson comorbidity index	1.27(1.12-1.42)	**0.0003**	1.19(1.02-1.36)	**0.02**	1.70(1.33-2.15)	**<0.0001**
Taiwanese vs Japanese	2.39(1.24-4.58)	**0.008**				
LVESVi per 10ml/m^2	1.36(1.10-1.65)	**0.003**	1.25(0.98-1.57)	0.06	1.93(1.20-3.10)	**0.006**

Sex was not associated with all-cause death univariably thus was not included in the models.
*All-cause mortality (n=63) occurred in 50 Taiwanese and 13 Japanese, respectively.

© 2023 Yang LT et al. *JAMA Network Open*.

eTable 7. Comparison between Asian and Western Individuals*

	**Asians, n=1259**	**Westerners, n=1072***
**Baseline demography**		
Age, year	64±17	59±18
Female	325(26)	189(18)
Body surface area, m^2	1.67±0.21	2.0±0.2
Charlson index	1.28±1.71	1.06 ± 1.25
Diastolic blood pressure, mmHg	65±13	64±13
NYHA† (1245/1050)	700(56)	675(64)
I		
II	418(34)	267(25)
III+IV	127(10)	108(10)
BAV fusion, RL fusion	177(73)	308(82)
RN	50(21)	63(17)
LN	15(6)	6(1)
LVEF, %	55±11	59±9
LVEDD, mm	60±8	60±7
LVESD, mm	41±9	40±7
LVEDDi, mm/m^2	36.6±5.2	30.3±4.1
LVESDi, mm/m^2	24.7±5.7	20.2±3.8
LVESDi>25 mm/m^2	481(38)	107(10)
LVEDVi, ml/m^2(n=1247/1007)	108±39	103±31
LVESVi, ml/m^2(n=1245/1007)	50±28	44±20
Annulus, mm	23.4±3.2	26±3
Indexed Annulus	14.2±1.9	13.0±1.6
Sinus of Valsalva, mm	40.5±8.2	41±6
Indexed Sinus of Valsalva	24.4±4.9	20.3±3.2
Mid-ascending aorta, mm	40.4±8.1	41±7
Indexed Mid-ascending aorta	24.7±5.5	20.4±4.4
**Surgical indications (481‡/552)**		
Symptoms	285(59)	345(63)
LVEF<50%	47(10)	23(4)
LVESD(i) >50mm(25mm/m^2)	75(16)	21(3.8)
Aortic aneurysm	21(4)	59(11)
LVEDD>65mm	36(7)	62(11)
Early surgery	17(4)	42(8)
Aortic valve repair	24(5)	122(22)
Bioprosthesis	330(72)	234(54)
Concomitant aorta surgery	170(35)	175(32)

© 2023 Yang LT et al. *JAMA Network Open*.

Concomitant coronary artery bypass grafting	48(10)	86(16)
8-year incidence of aortic valve surgery§	48±2%	55±2%
8-year survival of total follow-up§	74±2%	78±2%
8-year survival under medical surveillance§	68±2%	72±2%
8-year post-AVS survival§	85±3%	84±2%

See Table 1 for abbreviations.

*Western cohort was from Yang LT et al. J Am Coll Cardiol. 2020 Jul 21;76(3):233-246.

†Patients with undetermined NYHA status were excluded.

‡Of 483 patients having aortic valve surgery, 2 had it performed elsewhere hence surgical indications were lacking.

§For ease of comparison, data at 8-year was shown.

© 2023 Yang LT et al. *JAMA Network Open*.
