# Supplementary material for: Analysis of Left Ventricular Indexes and Mortality Among Asian Adults With Hemodynamically Significant Chronic Aortic Regurgitation
Source: JAMA Netw Open. 2023 Mar 24;6(3):e234632. doi: 10.1001/jamanetworkopen.2023.4632 (PMC10313151; doi:10.1001/jamanetworkopen.2023.4632)
Supplement: Supplement 2. — Data Sharing Statement [file jamanetwopen-e234632-s002.pdf]

Data Sharing Statement

Yang. Analysis of Left Ventricular Indexes and Mortality Among Asian Adults With Hemodynamically Significant Chronic Aortic Regurgitation. *JAMA Netw Open*. Published March 24, 2023. doi:10.1001/jamanetworkopen.2023.4632

Data
Data available: No
